# Prognostic Value of Coronary Microvascular Dysfunction Assessed by Coronary Angiography–Derived Index of Microcirculatory Resistance in Patients With ST‐Segment Elevation Myocardial Infarction

**DOI:** 10.1002/clc.24318

**Published:** 2024-07-08

**Authors:** Han Chao, Gao Jun‐Qing, Zhang Hong, Qi Zhen, Zhang Hui, An Wen, Yang Chenghao, Zhang Ling‐Xiao, Chen Shuang‐Yu, Liu Zong‐Jun

**Affiliations:** ^1^ Department of Cardiology, Putuo Hospital Shanghai University of Traditional Chinese Medicine Shanghai China; ^2^ Suzhou RainMed Medical Technology Co. Ltd. Suzhou China

**Keywords:** coronary angiography–derived index of microcirculatory resistance, coronary microvascular dysfunction, major adverse cardiac events, ST‐segment elevation myocardial infarction

## Abstract

**Background:**

CaIMR is proposed as a novel angiographic index designed to assess microcirculation without the need for pressure wires or hyperemic agents. We aimed to investigate the impact of caIMR on predicting clinical outcomes in STEMI patients.

**Methods:**

One hundred and forty patients with STEMI who received PCI in Putuo Hospital of Shanghai from October 2021 to September 2022 were categorized into CMD and non–CMD groups according to the caIMR value. The baseline information, patient‐related examinations, and the occurrence of MACE at the 12‐month follow‐up were collected to investigate risk factors in patients with STEMI.

**Results:**

We divided 140 patients with STEMI enrolled into two groups according to caIMR results, including 61 patients diagnosed with CMD and 79 patients diagnosed with non–CMD. A total of 21 MACE occurred during the 1 year of follow‐up. Compared with non–CMD group, patients with CMD showed a significantly higher risk of MACE. A multivariate Cox regression model was conducted for the patients, and it was found thatcaIMR was a significant predictor of prognosis in STEMI patients (HR: 8.921). Patients with CMD were divided into culprit vascular CMD and non‐culprit vascular CMD, and the result found that culprit vascular CMD was associated with the incidence of MACE (OR: 4.75) and heart failure (OR: 7.50).

**Conclusion:**

CaIMR is a strong predictor of clinical outcomes and can provide an objective risk stratification for patients with STEMI. There is a strong correlation among leukocyte index, the use of furosemide, Killips classification, and clinical outcomes.

## Graphical abstract

The present study represents a single‐center retrospective observational investigation involving patients admitted to Putuo Hospital of Shanghai for ST‐segment elevation myocardial infarction (STEMI) between October 2021 and September 2022. All the patients completed emergency PCI at admission. A comprehensive follow‐up of all patients was conducted with a duration of 12 months. This study investigated the correlation between caIMR and the prognosis of patients with STEMI after PCI. The major findings of the present study were as follows: (1) the incidence of MACE in CMD patients was higher than that in non–CMD patients; (2) caIMR, leukocyte, furosemide, and Killips classification were independent predictors of prognosis in patients with STEMI; (3) patients with CMD were divided into culprit vascular CMD and non‐culprit vascular CMD. Culprit vascular CMD was associated with the incidence of a heart failure event, while non‐culprit vascular CMD was irrelevant to the occurrence of any MACE. Our findings provide valuable insight into the impact of caIMR on STEMI patients, given its non‐invasiveness, pressure‐wire–free design, and no hyperemic agents. (a) Coronary microvascular dysfunction after PCI in patients with STEMI, (b) acquisition of two angiographic images of the target vessels with a projection angle >30°, (c) reconstruction of three‐dimensional blood vessels, and (d) the formula of caIMR calculation.

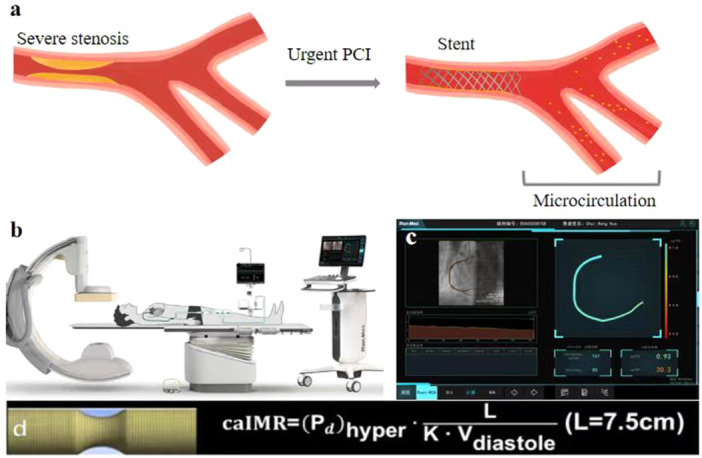

## 1 Introduction

ST‐segment elevation myocardial infarction (STEMI) stands as a serious threat to human health [1]. Although percutaneous coronary intervention (PCI) can restore the blood flow of the epicardial coronary artery, coronary microvascular dysfunction (CMD) still exists in patients with STEMI [2]. Nevertheless, residual risk endures even in those patients who have successfully undergone reperfusion of the culprit vessels following PCI [3]. Among the vital factors accounting for this phenomenon lies CMD, characterized by impaired vascular function, microvascular obstruction (MVO), microvascular injury (MVI), and intramyocardial hemorrhage (IMH), to name a few [4]. Recently, it has come to light that the incidence of CMD after PCI can soar up to an astonishing 50%, leading to undesirable clinical outcomes [5, 6]. These compelling findings have shed light on the significance of coronary microcirculation function to further improve the prognosis of STEMI.

The index of microcirculatory resistance (IMR) is a physiologic index determined using a pressure wire, enabling a quantitative evaluation of microcirculatory function within a specific vessel territory. However, conventional IMR measurement requires a pressure–temperature sensor wire and induction of hyperemia, which limits the adoption of IMR in clinical practice. Coronary angiography–derived IMR (caIMR) represents a novel angiographic index designed to assess microcirculation without the need for pressure wires or hyperemic agents. It offers an alternative approach to the traditional wire‐based IMR for prognostic stratification. The use of caIMR eliminates the need for additional catheterization, which is particularly advantageous in cases of STEMI and substantially reduces the risk of vascular complications associated with catheterization. Extensive research has been conducted to ensure the accuracy of caIMR. In a retrospective validation study, caIMR demonstrated a strong correlation with wire‐based IMR in a cohort of 56 patients with ischemia and non‐obstructive coronary artery disease (INOCA) [7]. This finding was subsequently confirmed in a recent prospective, multicenter, randomized study known as the FLASH IMR study [8].

In this study, we sought to evaluate the prognostic impact of coronary microvascular function derived by caIMR in STEMI patients.

## 2 Methods

### 2.1 Study Population

The present study represents a single‐center retrospective observational investigation involving patients admitted to Putuo Hospital of Shanghai for STEMI between October 2021 and September 2022. All the patients completed emergency PCI at admission. A comprehensive follow‐up of all patients was conducted with a duration of 12 months. Inclusion criteria encompassed patients who met the following conditions: (1) age over 18 years; (2) clinical diagnosis of STEMI; and (3) received emergency PCI treatment. Exclusion criteria included patients who met any of the following conditions: (1) residual stenosis of the culprit vessel equal to or greater than 70% following PCI; (2) systolic blood pressure of 90 mmHg or lower; and (3) coronary arteriography revealing the presence of a myocardial bridge in either the culprit or non–culprit vessels. Specifically for caIMR, exclusion criteria encompassed cases with inadequate contrast opacification, significant vascular overlap or distortion of the target vessel, and poor angiographic image quality, which rendered contour detection, as required by the FLASH software, unfeasible.

The study meticulously documented the fundamental clinical information, previous medical history, and laboratory data of all subjects. Additionally, data concerning electrocardiograms, echocardiography, and coronary angiography were collected from all participants.

The ethics committee of our esteemed institution bestowed approval upon this investigation, while concurrently ensuring that all enrolled patients formally acknowledged their informed consent by affixing their signatures (Ethics No.: PTEC‐A‐2021‐21‐1).

### 2.2 Measurement of caIMR

caIMR was calculated by a skilled engineer who was blind to the clinical data. To accurately obtain the length of target vessels, two angiographic images of the target vessels with a projection angle > 30° were used to rebuild a three‐dimensional vessel. Invasive aortic blood pressure was reviewed and input into the console. The result of the caIMR was measured with FlashAngio software (Rainmed Ltd.) with a proprietary fluid dynamic algorithm that was described earlier [7]. The formula of caIMR calculation is as follows: caIMR = $Pd_{\mathrm{hyp}}\frac{L}{K\cdot V_{\mathrm{diastole}}}$, where *Pd*_hyp_ is the mean invasive blood pressure, *L* is the length of the target vessel measured through a three‐dimensional vessel's model, *V*_diastole_ is the mean coronary flow velocity assessed by the frame counts, and *K* is a constant. We also recorded the coronary angiography–derived fractional flow reserve (caFFR) from the FlashAngio system. The evaluation of caFFR and caIMR was conducted as described in previous studies [9, 10]. CMD was defined as culprit vessel caIMR > 40 U [11] and non–culprit vessel caIMR > 25 U [12].

### 2.3 Follow‐Up and Endpoint of the Study

All participants were followed up for 1 year via outpatient visits, inpatient chart reviews, or telephone interviews to determine their clinical status. We requested our patients to take the outpatient follow‐up termly to assess their clinical status. MACE was the primary endpoint of the present study, defined as composite events of cardiovascular death, readmission for heart failure, reinfarction, and revascularization. All deaths were considered cardiac unless a definite noncardiac cause was documented. Heart failure was defined as the emergence of new or exacerbated clinical signs and symptoms of heart failure, supported by noninvasive imaging findings or an elevated concentration of N‐terminal pro–B‐type natriuretic peptide (NT‐proBNP), and a discharge diagnosis of congestive heart failure. Revascularization was defined as the re‐intervention of a previous vessel or emerging stenosis requiring intervention.

### 2.4 Statistical Analysis

The Kolmogorov–Smirnov test was used to determine the distribution of numerical data. Variables with a normal distribution are presented as the mean ± standard deviation and compared using the independent samples *t*‐test. Variables with skewed distribution are reported as the median with interquartile range (IQR) and were compared using the nonparametric Mann–Whitney test. The categorical data were expressed as use cases and percentages and were tested using *χ* ^2^ or Fisher's exact tests. Bivariate correlation analysis was used to evaluate the relationship between the variables. A univariate Cox regression model was established and variables with *p* < 0.1 were entered into the multivariate analysis. Clinical factors (age, sex, current smoking, and diabetes) were also included. We then used a stepwise algorithm to investigate independent determinants of patient prognosis in a multivariate Cox regression analysis and incorporated significant variables into the final prediction model. Kaplan–Meier event‐free curves were plotted for CMD and non–CMD patients, and the difference between the two curves was tested using log rank. The correlation between different blood vessels and cardiac adverse events was studied by Pearson's correlation analysis. A two‐sided *p* value < 0.05 was considered statistically significant. All analyses were performed using R version 4.0.5 (The R Foundation for Statistical Computing, Vienna, Austria) and SPSS 25.0 (IBM Corp, Armonk, NY, USA).

## 3 Results

**Figure 1:**
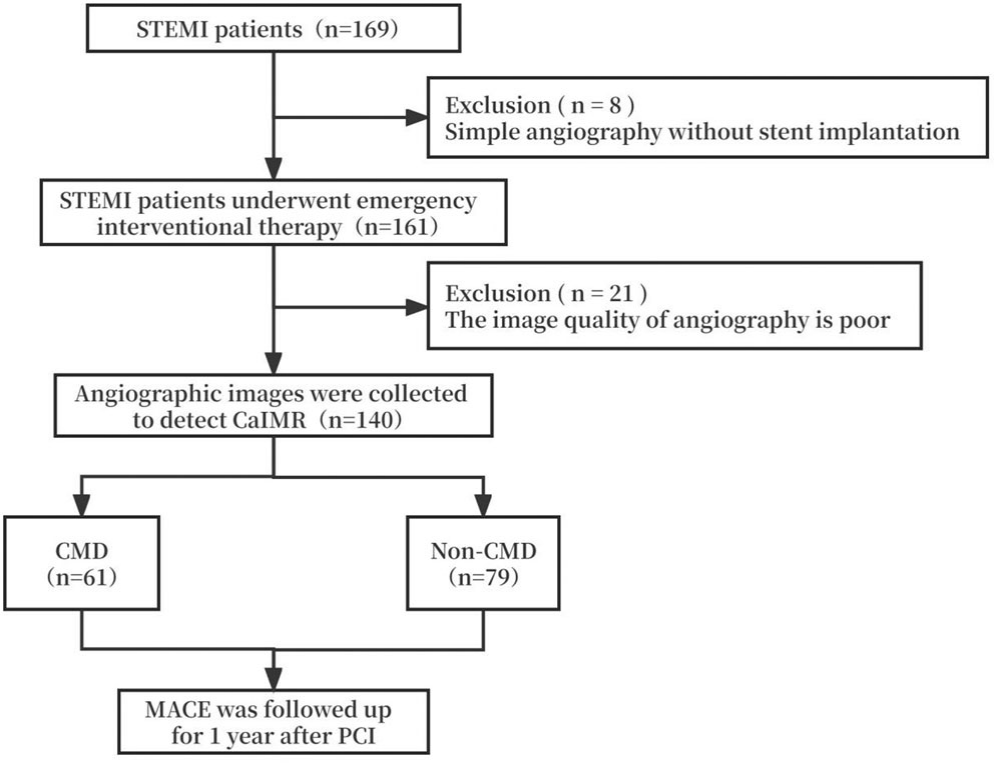
Flowchart of the study selection process. CMD, coronary microvascular dysfunction; MACE, major adverse cardiac events.

Figure 1 shows the flowchart of this study. A total of 169 patients with STEMI were initially screened, and 140 patients satisfied the inclusion criteria and were finally enrolled during a follow‐up time of 1 year. Patients were then divided into the CMD group (*n* = 61) and the non–CMD group (*n* = 79) for comparisons of baseline characteristics.

### 3.1 Baseline Characteristics of the Study Population

As shown in Table 1, the mean age of the patients was 64.5 years, 81.4% were male, 49.3% had complications due to hypertension, and 50.0% had a history of smoking. As measured by caIMR, there were 61 (43.6%) patients with CMD (culprit vessel caIMR > 40 U and/or non–culprit vessel caIMR > 25 U) and 79 (56.4%) patients without CMD. In the detection of heart‐related indicators, it can be found that the levels of BNP, cTnI, and CK in CMD patients are higher than those in non–CMD patients, especially the peak of cTnI, and there are statistically significant differences between the two groups. In terms of medication, all patients without contraindications were treated with aspirin, P2Y12 inhibitors, and statins, and more than 60% of patients were treated with β‐blockers and ACEI/ARB/ARNI drugs when discharged. There were more than 10 patients with cardiac dysfunction. Furosemide, antisterone, and other diuretics were used to relieve myocardial load during hospitalization. During the follow‐up period, all patients were still taking aspirin, P2Y12 inhibitors, and statins. More than 80% of patients were treated with β‐blockers and ACEI/ARB/ARNI drugs. Only a few patients diagnosed with heart failure use diuretics.

**Table 1:** Characteristics of the study population and clinical outcomes at 12 months.

	Total (*n* = 140)	Non–CMD (*n* = 79, 56.4%)	CMD (*n* = 61, 43.6%)	*p* value
General characteristics				
Age (years)	64.5 (57.0–70.0)	65.0 (57.5–71.5)	64.5 (56.0–58.0)	0.624
Male, *n* (%)	114 (81.4)	64 (81.0)	50 (82.0%)	0.885
BMI (kg/m^2^)	24.2 ± 3.1	24.2 ± 3.1	24.1 ± 3.2	0.970
Vascular recanalization time				
Time from onset to hospital arrival (h)	4.98 ± 1.09	4.86 ± 0.99	5.14 ± 1.19	0.35
Door‐to‐Balloon time (min)	66.18 ± 18.25	66.2 ± 18.53	66.15 ± 18.26	0.99
Cardiovascular risk factors				
Hypertension	69 (49.3%)	38 (48.1%)	31 (50.8%)	0.750
Diabetes	37 (26.4%)	21 (26.6%)	16 (26.2%)	0.963
Hyperlipidemia	6 (4.3%)	5 (6.3%)	1 (1.6%)	0.348
Chronic kidney disease^a^	2 (1.4%)	1 (1.3%)	1 (1.6%)	1.000
Current smoking	70 (50.0%)	39 (49.4%)	31 (50.8%)	0.865
Previous of AMI	9 (6.4%)	4 (5.1%)	5 (8.2%)	0.688
Previous CABG	1 (0.7%)	1 (1.3%)	0 (0.0%)	0.564
LVEF (%)	53.26 ± 8.84	52.75 ± 8.77	52.61 ± 9.88	0.502
Heart‐related laboratory profiles				
BNP (peak) (pg/mL)	632.0 (342.9–998.5)	556.0 (346.8–994.5)	655.5 (319.0–996.0)	0.646
cTnI (peak) (ng/mL)	58.6 (21.7–132.8)	24.7 (3.2–161.2)	132.5 (29.7.1–280.1)	0.030
CK (peak) (ng/mL)	161.2 (55.5–300.0)	131.4 (55.2–290.1)	200.9 (66.8–300.0)	0.072
BNP (seventh day) (pg/mL)	276.4 (115.7–570.5)	229.4 (127.3–490.0)	320.0 (115.8–642.5)	0.124
cTnI (seventh day) (ng/mL)	1.86 (0.39–6.1)	1.84 (0.48–5.05)	3.37 (0.45–6.22)	0.761
CK (seventh day) (ng/mL)	2.29 (1.51–3.98)	2.19 (1.62–3.72)	2.50 (1.55–4.61)	0.517
Cardiac function (Killip class)				
I	128 (91.4%)	72 (91.1%)	56 (91.8%)	0.889
II	3 (2.1%)	2 (2.5%)	1 (1.6%)	0.597
III	2 (1.4%)	2 (2.5%)	0 (0.0%)	0.317
IV	7 (5.0%)	3 (3.8%)	4 (6.6%)	0.359
Discharge medication				
Aspirin, P2Y12 inhibitors	140 (100%)	79 (100%)	61 (100%)	1.000
Statin	140 (100%)	79 (100%)	61 (100%)	1.000
β‐Blocker	101 (72.1%)	56 (70.9%)	45 (73.8%)	0.706
ACEI/ARB/ARNI	86 (61.4%)	49 (62.0%)	37 (60.7%)	0.869
Antisterone	46 (32.9%)	27 (34.2%)	19 (31.1%)	0.705
Furosemide	46 (32.9%)	28 (35.4%)	18 (29.5%)	0.458
Follow‐up 1‐year medication				
Aspirin, P2Y12 inhibitors	140 (100%)	79 (100%)	61 (100%)	1.000
Statin	140 (100%)	79 (100%)	61 (100%)	1.000
β‐Blocker	117 (83.6%)	68 (86.1%)	49 (80.3%)	0.363
ACEI/ARB/ARNI	114 (81.4%)	64 (81.0%)	50 (82.0%)	0.885
Antisterone	21 (15.0%)	12 (15.19%)	9 (14.8%)	0.943
Furosemide	21 (15.0%)	12 (15.19%)	9 (14.8%)	0.943
Culprit vessel				
LAD	84 (60.0%)	50 (63.3%)	34 (55.7%)	0.366
LCX	14 (10.0%)	7 (8.9%)	7 (11.5%)	0.609
RCA	42 (30.0%)	22 (27.8%)	20 (32.8%)	0.527
Multiple vessel disease	102 (75%)	61 (79.2%)	41 (69.5%)	0.194
Glycoprotein IIb/IIIa inhibitor use	94 (67.1%)	54 (68.4%)	40 (65.6%)	0.728
Thrombus aspiration	48 (34.3%)	24 (30.4%)	24 (39.3%)	0.268
Mean stent diameter (mm)	3.00 (2.69–3.11)	3.00 (2.75–3.00)	3.00 (2.68–3.38)	0.718
Total length of stents (mm)	30.0 (22.0–43.0)	30.0 (23.0–46.0)	26.0 (22.0–34.0)	0.067
Symptom to reperfusion (h)	3.3 (2.2–6.4)	3.5 (2.3–6.3)	3.9 (2.5–7.4)	0.820
Clinical outcomes at 12 months according to caIMR				
Cardiovascular death	8 (5.71%)	3 (3.8%)	5 (8.2%)	—
Reinfarction	1 (1.3%)	1 (1.3%)	0 (0)	—
Heart failure	15 (10.71%)	5 (6.3%)	10 (16.4%)	—
Revascularization	2 (1.43%)	1 (1.3%)	1 (1.64%)	—
Total MACE	21 (15%)	7 (8.9%)	14 (22.9%)	—

Abbreviations: BMI, body mass index; BNP, brain natriuretic peptide; CK, creatine kinase; cTnI, cardiac troponin I; LAD, left anterior descending coronary artery; LCx, left circumflex artery; LVEF, left ventricular ejection fraction; MACE, major adverse cardiac events; cardiovascular death as death due to malignant arrhythmia, myocardial infarction, or refractory heart failure; RCA, right coronary artery.

^a^ Chronic kidney disease was defined as estimated glomerular filtration rate < 60 mL/min/m^2^ or serum creatinine > 1.4 mg/dL.

Through further analysis of the data related to coronary intervention (Table 1), the culprit vessel can be found among the patients with STEMI, LAD was found in 84 cases, LCX in 14 cases, and RCA in 42 cases. Intraoperative use of glycoprotein IIb/IIIa inhibitors, thrombus aspiration, and other treatments was not significantly different between the two groups nor were the mean diameter and total length of implanted stents significantly different.

### 3.2 Clinical Outcome

**Figure 2:**
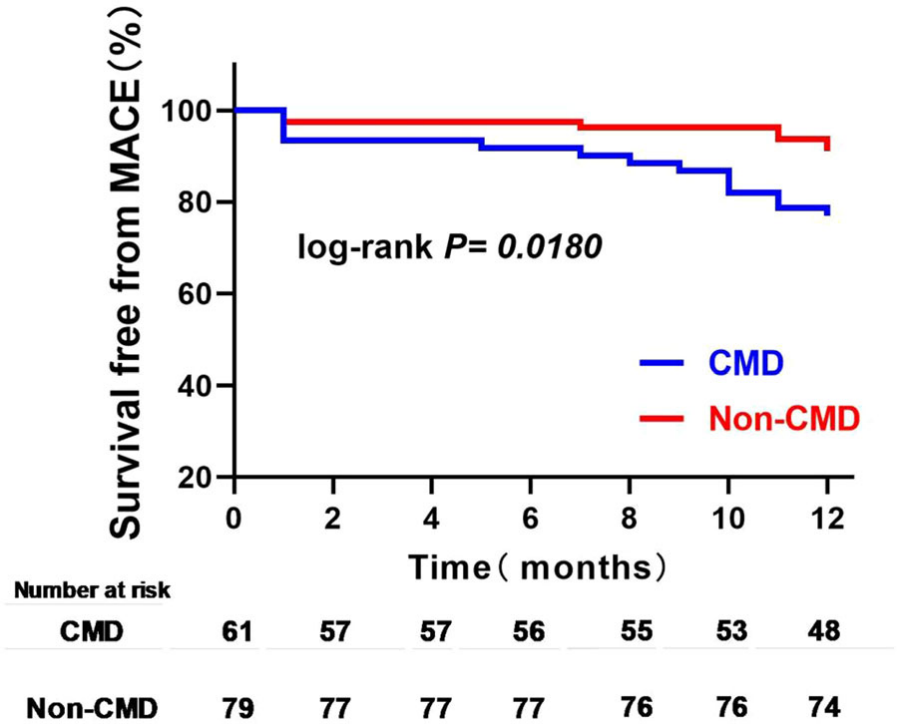
Kaplan–Meier survival curves for MACE in CMD patients versus non–CMD patients.

In the 12‐month follow‐up after PCI, the incidence of total MACE in CMD patients was significantly higher than that in non–CMD patients (22.9% vs. 8.9%; *p* = 0.021) (Table 1). Kaplan–Meier survival curves also demonstrated a significantly high MACE in CMD patients than in non–CMD patients (log rank *p* = 0.0180) (Figure 2). Patients with CMD had a higher risk of MACE than those non–CMD (HR: 2.821, 95% CI: 1.138–6.993, *p* = 0.025). These results validated obviously that caIMR has a good predictive value in STEMI patients.

### 3.3 Outcome Predictors

Table 2 displays the univariable and multivariable predictors of MACE. Cox regression analysis shows that some indexes such as caIMR, leukocyte, CRP, BNP (peak), cTnI (seventh day), LVEF, furosemide/antisterone use, diabetes, and Killips classification were important prognostic indicators of patients with STEMI. Then, we included postoperative caIMR, leukocyte, BNP, cTnI, furosemide, and other important predictors for multivariate analysis. In the multivariate Cox regression model, it was found that caIMR (HR: 8.921, 95% CI: 2.456–32.399, *p* = 0.001), leukocyte (HR: 1.166, 95% CI: 1.018–1.336, *p* = 0.026), furosemide (HR: 32.390, 95% CI: 2.088–502.524, *p* = 0.013), Killips classification (HR: 1.725, 95% CI: 1.069–2.782, *p* = 0.0025) were independent predictors of prognosis in patients with STEMI (Table 2).

**Table 2:** Predictors of clinical outcome.

	Univariate analysis		Multivariate analysis	
	HR (95% CI)	*p* value	HR (95% CI)	*p* value
caIMR	2.821 (1.138–6.993)	0.025	8.921 (2.456–32.399)	0.001
Leukocyte	1.111 (1.014–1.218)	0.024	1.166 (1.018–1.336)	0.026
CRP	1.011 (1.004–1.019)	0.004		
BNP (peak)	1.000 (1.000–1.001)	0.027		
BNP (seventh day)	1.001 (1.000–1.001)	0.052		
cTnI (seventh day)	1.008 (1.002–1.014)	0.008		
LVEF	0.949 (0.910–0.989)	0.014		
ACEI/ARB/ARNI	0.436 (0.184–1.035)	0.060		
Antisterone	2.948 (1.242–6.998)	0.014		
Furosemide	3.691 (1.529–8.910)	0.004	32.390 (2.088–502.524)	0.013
Diabetes	2.709 (1.150–6.383)	0.023		
Killips classification	1.893 (1.333–2.689)	< 0.001	1.725 (1.069–2.782)	0.025
Post–PCI TIMI	0.477 (0.236–0.965)	0.039		

**Figure 3:**
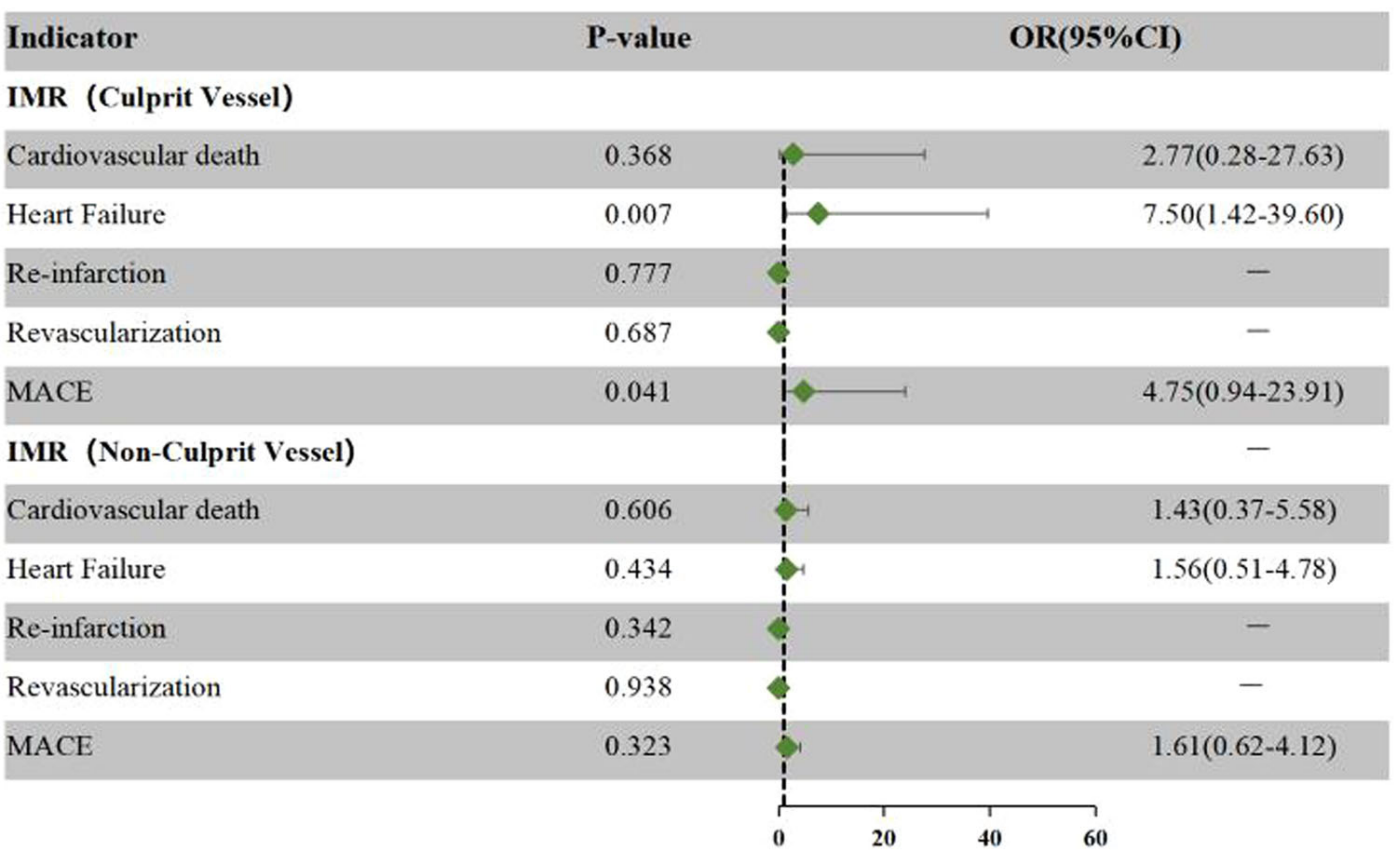
Correlation between different vessels and MACE. The green symbols represent the OR values between IMR and MACEs.

During the 12‐month follow‐up after PCI, we conducted correlation analysis on caIMR of different vessels and MACE during the 1 year of follow‐up. We found that culprit vessel caIMR > 40 U is significantly correlated with heart failure events (OR: 7.50, 95% CI: 1.42–39.60, *p* = 0.007). For non–culprit vessels, caIMR > 25 U was irrelevant to the occurrence of MACE (Figure 3).

## 4 Discussion

STEMI is a grave manifestation of coronary heart disease, and it stands as a prominent contributor to morbidity and mortality [13]. Currently, early revascularization serves as the principal treatment for patients with STEMI. Research has established a direct correlation between myocardial perfusion and the magnitude of infarct size, left ventricular remodeling, and mortality subsequent to PCI [14, 15]. It is widely believed that compromised myocardial perfusion predominantly arises from CMD in cases of preserved epicardial flow patency. Furthermore, comprehensive investigations have demonstrated the presence of CMD in non–culprit vessels among STEMI patients [16, 17, 18]. To embark on CMD therapy, we contend that prompt and precise assessment of CMD is indispensable in patients with STEMI.

With the advancement in angiography technology, caIMR is proposed as a novel pressure‐wire–free tool to assess CMD. This innovation effectively addresses several clinical challenges encountered in wire‐based IMR measurements, such as the necessity of vasodilator usage, repeated saline injections, prolonged measurement duration, and heig,htened surgical risks [11, 19, 20, 21, 22, 23]. In a comparative study with wire‐based IMR as the diagnostic standard, a strong correlation between IMR and caIMR was observed [8]. Further investigations have also demonstrated that caIMR has a high diagnostic sensitivity, specificity, and accuracy in CMD patients [24, 25]. Moreover, it has emerged as an influential independent predictor for prognostic risk assessment [11, 26]. A series of evidence have shown that caIMR has good diagnostic and predictive value.

In this cohort study, we estimated the sample size according to the incidence of MACE in patients with acute STEMI with microcirculation disorder and non‐microcirculation disorder in previous studies [19], and *α* = 0.05, 1 − *β* = 0.8. The results showed that the total number of samples should be greater than 74, and the ratio of the two groups should be about 1:1. In the sensitivity analysis, to minimize the impact of reverse causality, we repeated the analysis after excluding subjects with MACE in the first 2 months of follow‐up. The direct association between postoperative caIMR and MACE after PCI in STEMI patients did not change. Then, in the sensitivity analysis excluding postoperative oral diuretics and low LVEF, the above correlation did not change substantially. These findings suggest that the current results are unlikely to be attributed to reverse causality. Regarding the possible changes in the correlation between caIMR and MACE in other STEMI patients after PCI, we included the above high‐risk factors in the multivariate model for correction. The results showed that the association between postoperative caIMR and MACE was independent of other risk factors, indicating that microcirculation dysfunction was directly associated with MACE after PCI in STEMI patients. In addition, our study used survival curve, Cox regression, and Pearson's correlation analysis to show the association between caIMR and MACE after PCI in STEMI patients.

This study investigated the correlation between caIMR and the prognosis of patients with STEMI after PCI. The major findings of the present study were as follows: (1) the incidence of MACE in CMD patients was higher than that in non–CMD patients; (2) caIMR, leukocyte, furosemide, and Killips classification were independent predictors of prognosis in patients with STEMI; and (3) patients with CMD were divided into culprit vascular CMD and non‐culprit vascular CMD. Culprit vascular CMD was associated with the incidence of a heart failure event, while non‐culprit vascular CMD was irrelevant to the occurrence of any MACE. Our findings provide valuable insight into the impact of caIMR on STEMI patients, given its non‐invasiveness, pressure‐wire–free design, and no hyperemic agents.

It has been previously documented that the occurrence of CMD after PCI varies from 15% to 50% [27]. Moreover, it has been previously established that CMD is associated with a higher risk of cardiovascular events [28]. Consequently, comprehensive analyses were conducted to investigate the association between STEMI patient outcomes and caIMR. In this study, we found that among 140 patients with STEMI after PCI, 61 (43.6%) patients still had CMD even after PCI treatment to improve epicardial flow. At the 12‐month follow‐up, we found that the incidence of major adverse cardiac events in CMD patients was higher than that in non–CMD patients, which may be related to larger infarct size due to suboptimal myocardial perfusion, resulting in adverse left ventricular remodeling and increased mortality [5].

Then, we performed a Cox regression analysis of clinical cardiovascular events in the 12‐month follow‐up for all patients. In univariate analysis, caIMR, leukocyte, CRP, BNP (peak), cTnI (seventh day), LVEF, furosemide/antisterone use, diabetes, Killips classification, and post–PCI TIMI were important predictors of prognosis in patients with STEMI. Further, in multivariate analysis, we found that caIMR, leukocyte, furosemide use, and Killips classification were independent predictors of prognosis in patients with STEMI. Previous studies have confirmed that myocardial enzyme profile and drug use of heart failure during hospitalization are closely related to the prognosis of myocardial infarction [29, 30]. Li et al. [24] and Carrick et al. [6] showed that the increase of IMR after PCI could predict adverse clinical outcomes in patients with myocardial infarction. This study was consistent with previous findings that caIMR was an independent predictor of long‐term prognosis in patients with STEMI [10]. Previous studies have shown that leukocyte index can predict the incidence of heart failure in patients with coronary heart disease [31]. This study reached a similar conclusion that the leukocyte index can be regarded as an independent predictor of prognosis in patients with STEMI. The mechanism may be related to excessive inflammatory activation in the infarct area and further aggravate myocardial remodeling. The Killips classification is used to evaluate the cardiac function and reflect the clinical manifestations of heart failure, and the administration of furosemide as a treatment for heart failure [29, 30]. Exactly, the use of furosemide during hospitalization indicates the clinical manifestations of heart failure, which may be related to left ventricular remodeling caused by myocardial infarction, and therefore affect the prognosis of patients with STEMI. That is why furosemide and Killips classification are predictors of clinical outcomes among patients with STEMI in this study.

We divided CMD patients into culprit vascular CMD and non‐culprit vascular CMD and found that culprit vascular CMD was associated with the incidence of heart failure events and total MACE, while non‐culprit vascular CMD was irrelevant to any MACE. Hou et al. found that caIMR showed a strong ability to predict left ventricular function recovery at 3 months after PCI [32], which was consistent with our results. In patients with culprit vascular CMD, due to the presence of CMD after the restoration of culprit coronary flow, poor myocardial perfusion leads to the expansion and severity of myocardial infarction, and the risk of death and major adverse events also increases. These results further confirm the importance of caIMR in the prognosis assessment of patients with STEMI. Furthermore, considering that the cut‐off value of 25 U for diagnosing CMD in non–culprit vessels stems from data derived from non‐STEMI cases, the predictive value of CMD in non–culprit vessels for STEMI may be compromised. Further research in subsequent stages is warranted to validate this hypothesis.

## 5 Limitations

The study has potential limitations. First, our study should be interpreted with caution in terms of a retrospective‐observational study, a single center, a small sample size, and a short follow‐up. Second, the relatively small number of MACE during the follow‐up period may have affected the statistical power of the study and limited the conclusions. Finally, we believe that the caIMR threshold used as a judgment of CMD in non‐culprit vascular needs to be revalidated in STEMI cases, taking into account some of the short‐term influencing factors in the acute phase of STEMI. These shortcomings will be further improved in future work.

## 6 Conclusions

CaIMR has a significant impact on the prognosis of patients with STEMI after PCI. It may be applied as an important indicator to predict the prognosis of patients with STEMI after PCI and also provides a strong theoretical basis for further improving the prognosis of patients with STEMI.

## Conflicts of Interest

The authors declare no conflicts of interest.

## Data Availability

Research data are not shared.
